# Supplementary material for: Multifactorial analysis of temperature, solute-to-solvent ratio, and ultrasound amplitude on the extraction of phenolic and antioxidant compounds from Aloysia citriodora Palau leaves
Source: PeerJ. 2025 Aug 19;13:e19821. doi: 10.7717/peerj.19821 (PMC12372784; doi:10.7717/peerj.19821)
Supplement: Supplemental Information 2 [file peerj-13-19821-s002.pdf]

Figura 2. Fitting Model of Flavonoids as a Function of Temperature, Solute/Solvent Ratio, and Amplitude

Flavonoids (mg Eq. Quercetin/g extract) Leverage Residuals

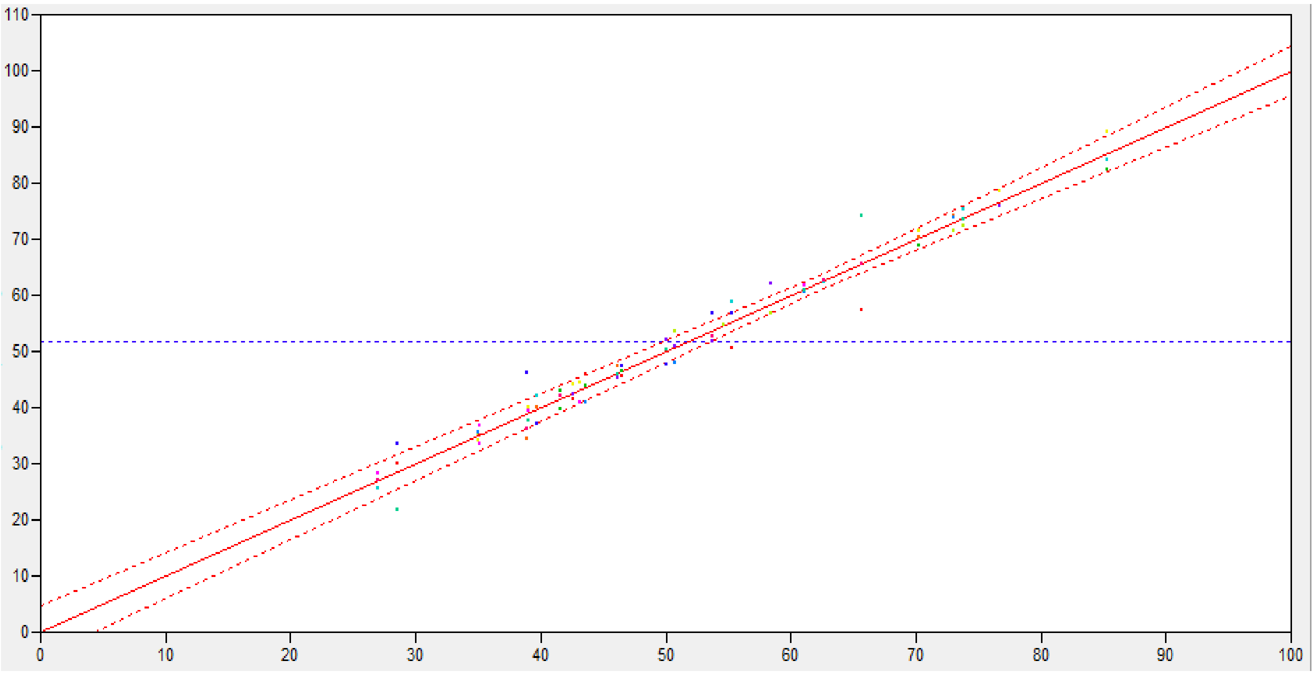

Temperature X Solute/Solvent X Amplitude Leverage, p<.0001

Equation 3. Prediction of Hydroxyl Radical (mg/mL)

$$
\begin{aligned}
\text{IC50 Hydroxyl Radical (mg/mL)} \\
&= 2.32 + a_1(\text{Temperature}) + a_2(\text{Solute/Solvent}) + a_3(\text{Amplitude}) \\
&+ a_4\,(\text{Temperature x Solute/Solvent}) + a_5\,(\text{Temperature x Amplitude}) \\
&+ a_6\,(\text{Solute/Solvent x Amplitude}) \\
&+ a_7\,(\text{Temperature x Solute/Solvent x Amplitude})
\end{aligned}
$$
