# Supplementary material for: Multifactorial analysis of temperature, solute-to-solvent ratio, and ultrasound amplitude on the extraction of phenolic and antioxidant compounds from Aloysia citriodora Palau leaves
Source: PeerJ. 2025 Aug 19;13:e19821. doi: 10.7717/peerj.19821 (PMC12372784; doi:10.7717/peerj.19821)

Figura 3. Fitting Model of IC50 Hydroxyl Radical as a Function of Temperature, Solute/Solvent Ratio, and Amplitude

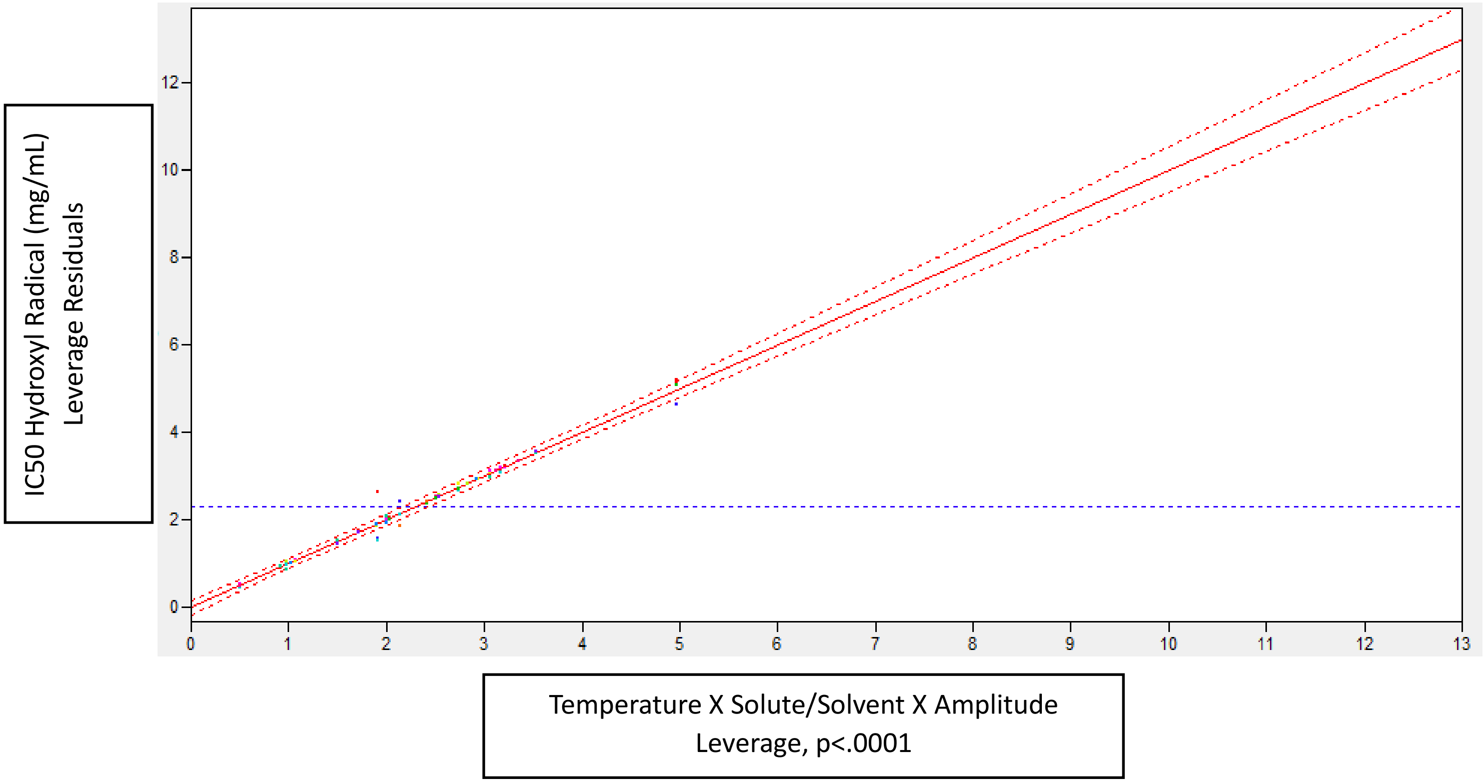

Supplement: Supplemental Information 3 [file peerj-13-19821-s003.pdf]
