# Supplementary material for: Multifactorial analysis of temperature, solute-to-solvent ratio, and ultrasound amplitude on the extraction of phenolic and antioxidant compounds from Aloysia citriodora Palau leaves
Source: PeerJ. 2025 Aug 19;13:e19821. doi: 10.7717/peerj.19821 (PMC12372784; doi:10.7717/peerj.19821)
Supplement: Supplemental Information 4 [file peerj-13-19821-s004.docx]

Supplementary Material

Equation 1. Prediction of Polyphenols (mg GAE/g extract)

$$Polyphenols (mg GAE/g extract) = 54.08 + a₁(\mathrm{Temperature}) + a₂(Solute/Solvent) + a₃(\mathrm{Amplitude}) + a₄ (Temperature x Solute/Solvent) + a₅ (Temperature x Amplitude) + a₆ (Solute/Solvent x Amplitude) + a₇ (Temperature x Solute/Solvent x Amplitude)$$

Table 1. Variables of the Effects of Individual and Combined Interactions for Predicting Polyphenols (mg GAE/g extract)

| Effects | variable by factor level |
| --- | --- |
| Temperature | Level 1: -21.21 Level 2: 6.29 Level 3: 14.91 |
| Solute/Solvent | Level 1: -3.84 Level 2: 4.54 Level 3: -0.69 |
| Amplitude | Level 1: -5.78 Level 2: 6.21 Level 3: -0.42 |
| Temperature 1 x Solute/Solvent Temperature 2 x Solute/Solvent Temperature 3 x Solute/Solvent | Level 1: 13.10 Level 2: 5.93 Level 3: -19.03  Level 1: 2.08 Level 2: -6.83 Level 3: 4.74  Level 1: 13.10 Level 2: 5.93 Level 3: -19.03 |
| Temperature 1 x Amplitude Temperature 2 x Amplitude Temperature 3 x Amplitude | Level 1: -4.45 Level 2: -1.77 Level 3: 6.23  Level 1: 9.41 Level 2: -9.97 Level 3: 0.56  Level 1: -4.95 Level 2: 11.75 Level 3: -6.79 |
| Solute/Solvent 1 x Amplitude Solute/Solvent 2 x Amplitude Solute/Solvent 3 x Amplitude | Level 1: 2.44 Level 2: -10.76 Level 3: 8.31  Level 1: -9.56 Level 2: 19.28 Level 3: -9.71  Level 1: 7.11 Level 2: -8.51 Level 3: 1.40 |
| Temperature 1 x Solute/Solvent 1 x Amplitude Temperature 1 x Solute/Solvent 2 x Amplitude Temperature 1 x Solute/Solvent 3 x Amplitude Temperature 2 x Solute/Solvent 1 x Amplitude Temperature 2 x Solute/Solvent 2 x Amplitude Temperature 2 x Solute/Solvent 3 x Amplitude Temperature 3 x Solute/Solvent 1 x Amplitude Temperature 3 x Solute/Solvent 2 x Amplitude Temperature 3 x Solute/Solvent 3 x Amplitude | Level 1: 9.18 Level 2: 2.31 Level 3:-11.49  Level 1: -2.53 Level 2: -13.97 Level 3: 16.51  Level 1: -6.65 Level 2: 11.66 Level 3:-5.01  Level 1: -8.17 Level 2: 14.55 Level 3: -6.38  Level 1: 15.27 Level 2: -7.17 Level 3:-8.08  Level 1: -7.09 Level 2: -7.37 Level 3: 14.47  Level 1: -1.01 Level 2: -16.86 Level 3: 17.87  Level 1: -12.73 Level 2: 21.15 Level 3: -8.41  Level 1: 13.75 Level 2: -4.28 Level 3: -9.46 |
