# Supplementary material for: Multifactorial analysis of temperature, solute-to-solvent ratio, and ultrasound amplitude on the extraction of phenolic and antioxidant compounds from Aloysia citriodora Palau leaves
Source: PeerJ. 2025 Aug 19;13:e19821. doi: 10.7717/peerj.19821 (PMC12372784; doi:10.7717/peerj.19821)
Supplement: Supplemental Information 5 [file peerj-13-19821-s005.docx]

Equation 2. Prediction of Flavonoids (mg Eq. Quercetin/g)

$$Flavonoids (mg Eq. Quercetin/g) extract) = 51.78 + a₁(\mathrm{Temperature}) + a₂(Solute/Solvent) + a₃(\mathrm{Amplitude}) + a₄ (Temperature x Solute/Solvent) + a₅ (Temperature x Amplitude) + a₆ (Solute/Solvent x Amplitude) + a₇ (Temperature x Solute/Solvent x Amplitude)$$

Table 2. Variables of the Effects of Individual and Combined Interactions for Predicting Flavonoids (mg Eq. Quercetin/g extract)

| Effects | variable by factor level |
| --- | --- |
| Temperature | Level 1: -28.49 Level 2: 19.26 Level 3: 9.22 |
| Solute/Solvent | Level 1: -7.77 Level 2: -4.56 Level 3: 12.33 |
| Amplitude | Level 1: -3.55 Level 2: -4.46 Level 3: 8.01 |
| Temperature 1 x Solute/Solvent Temperature 2 x Solute/Solvent Temperature 3 x Solute/Solvent | Level 1: 2.19 Level 2: 18.88 Level 3: -21.07  Level 1: 15.00 Level 2: -20.84 Level 3: 5.83  Level 1: -17.20 Level 2: 1.95 Level 3: 15.24 |
| Temperature 1 x Amplitude Temperature 2 x Amplitude Temperature 3 x Amplitude | Level 1: -2.57 Level 2: 4.35 Level 3: -1.77  Level 1: 11.42 Level 2: -10.43 Level 3: -0.98  Level 1: -8.84 Level 2: 6.07 Level 3: 2.76 |
| Solute/Solvent 1 x Amplitude Solute/Solvent 2 x Amplitude Solute/Solvent 3 x Amplitude | Level 1: -1.23 Level 2: -1.16 Level 3: 2.39  Level 1: -3.25 Level 2: 1.70 Level 3: 1.55  Level 1: 4.49 Level 2: -0.53 Level 3: -3.95 |
| Temperature 1 x Solute/Solvent 1 x Amplitude Temperature 1 x Solute/Solvent 2 x Amplitude Temperature 1 x Solute/Solvent 3 x Amplitude Temperature 2 x Solute/Solvent 1 x Amplitude Temperature 2 x Solute/Solvent 2 x Amplitude Temperature 2 x Solute/Solvent 3 x Amplitude Temperature 3 x Solute/Solvent 1 x Amplitude Temperature 3 x Solute/Solvent 2 x Amplitude Temperature 3 x Solute/Solvent 3 x Amplitude | Level 1: 21.97 Level 2: -9.16 Level 3: -12.81  Level 1: -16.72 Level 2: -1.71 Level 3: 18.43  Level 1: -5.24 Level 2: 10.87 Level 3: -5.62  Level 1: -24.81 Level 2: 21.28 Level 3: 3.52  Level 1: 33.44 Level 2: -23.25 Level 3: -10.18  Level 1: -8.63 Level 2: 1.96 Level 3: 6.66  Level 1: 2.83 Level 2: -12.12 Level 3: 9.28  Level 1: -16.71 Level 2: 24.96 Level 3: -8.24  Level 1: 13.88 Level 2: -12.84 Level 3: -1.03 |
