# Supplementary material for: Multifactorial analysis of temperature, solute-to-solvent ratio, and ultrasound amplitude on the extraction of phenolic and antioxidant compounds from Aloysia citriodora Palau leaves
Source: PeerJ. 2025 Aug 19;13:e19821. doi: 10.7717/peerj.19821 (PMC12372784; doi:10.7717/peerj.19821)
Supplement: Supplemental Information 6 [file peerj-13-19821-s006.docx]

Equation 3. Prediction of Hydroxyl Radical (mg/mL)

$$IC50 Hydroxyl Radical (mg/mL) = 2.32 + a₁(\mathrm{Temperature}) + a₂(Solute/Solvent) + a₃(\mathrm{Amplitude}) + a₄ (Temperature x Solute/Solvent) + a₅ (Temperature x Amplitude) + a₆ (Solute/Solvent x Amplitude) + a₇ (Temperature x Solute/Solvent x Amplitude)$$

Table 3. Variables of the Effects of Individual and Combined Interactions for Predicting IC50 of Hydroxyl Radical

| Effects | variable by factor level |
| --- | --- |
| Temperature | Level 1: 1.00 Level 2: -0.66 Level 3: -0.33 |
| Solute/Solvent | Level 1: 0.100 Level 2: -0.57 Level 3: 0.46 |
| Amplitude | Level 1: 0.93 Level 2: -0.62 Level 3: -3.10 |
| Temperature 1 x Solute/Solvent Temperature 2 x Solute/Solvent Temperature 3 x Solute/Solvent | Level 1: -1.55 Level 2: -0.92 Level 3: 2.48  Level 1: 0.35 Level 2: 0.63 Level 3: -0.98  Level 1: 1.20 Level 2: 0.28 Level 3: -1.49 |
| Temperature 1 x Amplitude Temperature 2 x Amplitude Temperature 3 x Amplitude | Level 1: 1.40 Level 2: -0.51 Level 3: -0.89  Level 1: -0.81 Level 2: 0.30 Level 3: 0.51  Level 1: -0.58 Level 2: 0.20 Level 3: 0.38 |
| Solute/Solvent 1 x Amplitude Solute/Solvent 2 x Amplitude Solute/Solvent 3 x Amplitude | Level 1: -0.68 Level 2: 0.53 Level 3: 0.14  Level 1: -0.27 Level 2: 0.08 Level 3: 0.19  Level 1: 0.95 Level 2: -0.61 Level 3: -0.34 |
| Temperature 1 x Solute/Solvent 1 x Amplitude Temperature 1 x Solute/Solvent 2 x Amplitude Temperature 1 x Solute/Solvent 3 x Amplitude Temperature 2 x Solute/Solvent 1 x Amplitude Temperature 2 x Solute/Solvent 2 x Amplitude Temperature 2 x Solute/Solvent 3 x Amplitude Temperature 3 x Solute/Solvent 1 x Amplitude Temperature 3 x Solute/Solvent 2 x Amplitude Temperature 3 x Solute/Solvent 3 x Amplitude | Level 1: -1.35 Level 2: 0.61 Level 3: 0.73  Level 1: -1.30 Level 2: 1.21 Level 3: 0.08  Level 1: 2.65 Level 2: -1.82 Level 3: -0.82  Level 1: 0.84 Level 2: -0.42 Level 3: -0.41  Level 1: 0.40 Level 2: -0.60 Level 3: 0.19  Level 1: -1.25 Level 2: 1.02 Level 3: 0.22  Level 1: 0.50 Level 2: -0.18 Level 3: -0.32  Level 1: 0.89 Level 2: -0.61 Level 3: -0.28  Level 1: -1.40 Level 2: 0.79 Level 3: 0.60 |
