# Supplementary material for: Multifactorial analysis of temperature, solute-to-solvent ratio, and ultrasound amplitude on the extraction of phenolic and antioxidant compounds from Aloysia citriodora Palau leaves
Source: PeerJ. 2025 Aug 19;13:e19821. doi: 10.7717/peerj.19821 (PMC12372784; doi:10.7717/peerj.19821)
Supplement: Supplemental Information 7 [file peerj-13-19821-s007.pdf]

Workflow Diagram of the Experimental Factorial Design

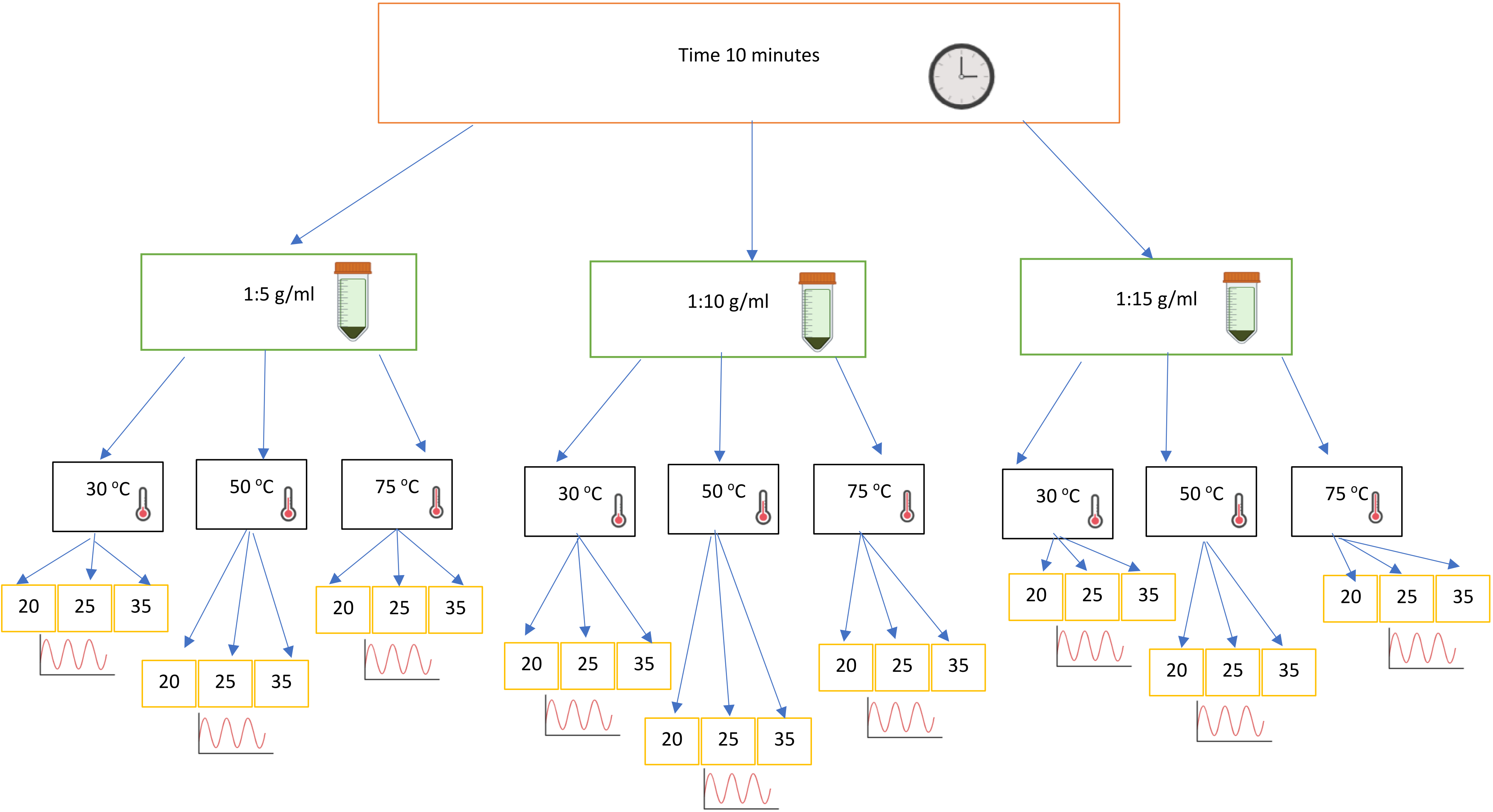

Orange box: time (min).

Green box: g of *A. citriodora* dried leaves/mL of solvent ratio. **R**

Black box: temperature (°C). **T**

Yellow box: amplitude (%). **A**
