# Supplementary material for: Multifactorial analysis of temperature, solute-to-solvent ratio, and ultrasound amplitude on the extraction of phenolic and antioxidant compounds from Aloysia citriodora Palau leaves
Source: PeerJ. 2025 Aug 19;13:e19821. doi: 10.7717/peerj.19821 (PMC12372784; doi:10.7717/peerj.19821)

Supplementary Material. Calibration *Curves for Total Phenolic Content and Total Flavonoid Content Determination*

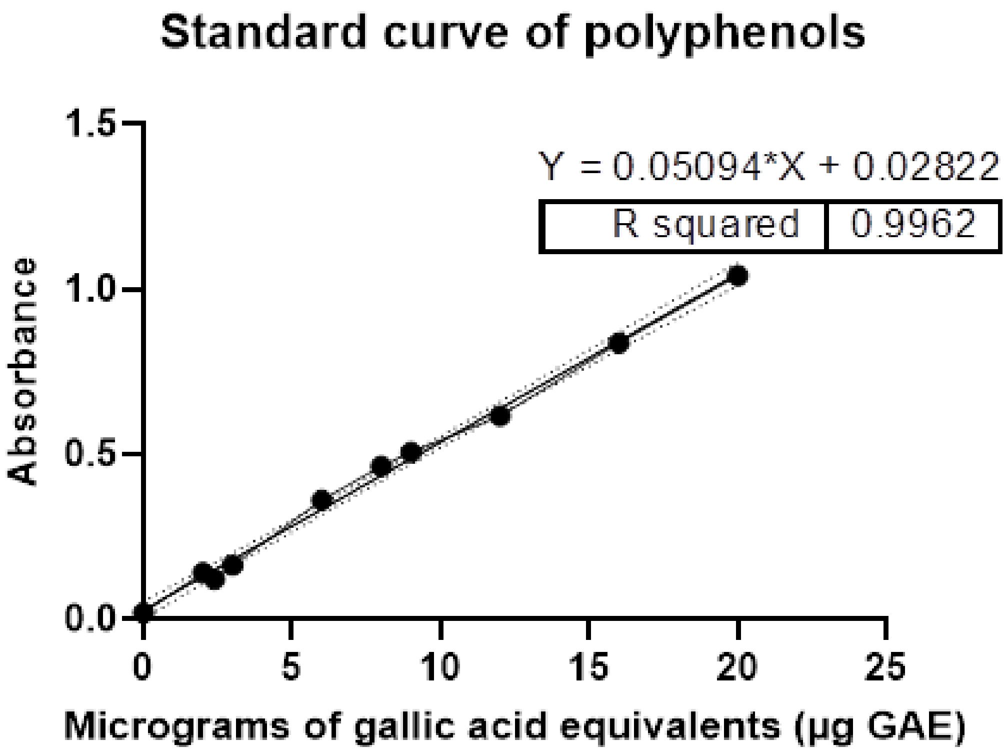

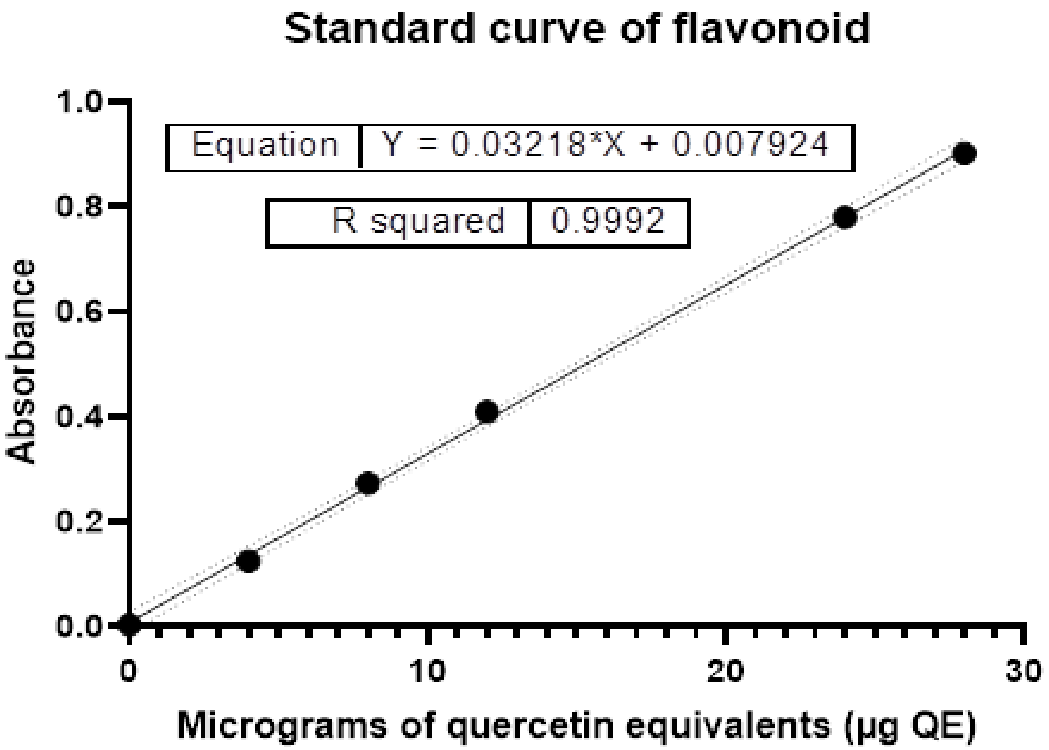

Supplement: Supplemental Information 8 [file peerj-13-19821-s008.pdf]
