# Supplementary material for: Multifactorial analysis of temperature, solute-to-solvent ratio, and ultrasound amplitude on the extraction of phenolic and antioxidant compounds from Aloysia citriodora Palau leaves
Source: PeerJ. 2025 Aug 19;13:e19821. doi: 10.7717/peerj.19821 (PMC12372784; doi:10.7717/peerj.19821)
Supplement: Supplemental Information 10 [file peerj-13-19821-s010.docx]

**Codebook**

**Pages 1 and 2 of the Excel file:**

Determination of solids present in the extracts

To quantify the amount of solids present in each extract, a drying procedure was carried out using pre-weighed ceramic capsules. Two replicates per extract were performed to ensure data repeatability.

Each capsule was weighed before and after drying the extract, allowing the calculation of the total solid content. From these values, an average in mg/mL was calculated, representing the concentration of solids in each extract.

This average value was later used to express the normalized results of antioxidant capacity and other analyzed properties.

**Page 3 of the Excel file:**

The table presents the results corresponding to the total solid concentration in the extracts (g/mL), as well as the amount of gallic acid equivalents (GAE), expressed in micrograms per gram of extract. Average values and standard deviations are also included to assess data variability and measurement precision.

**Page 4 of the Excel file:**

Similarly, the table shows the flavonoid content expressed in milligrams and micrograms of quercetin. The total solid concentration of the extract (g/mL) and the amount of quercetin per gram of extract, expressed in micrograms and milligrams, are also provided. Average values are reported to represent the central tendency of the measurements.

**Page 5 of the Excel file**:

The table presents the results of hydroxyl radical scavenging activity, including values in milligrams and the IC₅₀ determined in microliters, milliliters, and milligrams per milliliter (mg/mL). The IC₅₀ represents the concentration required to inhibit 50% of hydroxyl radical activity and serves as an indicator of antioxidant efficiency.

**Page 6 of the Excel file:**

Results of the DPPH radical scavenging assay are shown, including measurements in milligrams and IC₅₀ values expressed in microliters, milliliters, milligrams per milliliter (mg/mL), and micrograms per milliliter (µg/mL). The IC₅₀ indicates the concentration of extract needed to inhibit 50% of the DPPH radicals and is a standard parameter for evaluating antioxidant capacity.

**Page 7 of the Excel file:**

The table presents the results of the ABTS radical scavenging assay, including measurements in milligrams and IC₅₀ values expressed in microliters, milliliters, milligrams per milliliter (mg/mL), and micrograms per milliliter (µg/mL). Additionally, the Trolox Equivalent Antioxidant Capacity (TEAC) is reported, with IC₅₀ values based on dry leaf mass (mg and g), as well as TEAC values normalized per milligram and per gram of extract. TEAC per 100 grams of extract is also included.
